# Ocular Manifestations and Therapeutic Options in Patients with Familial Amyloid Polyneuropathy: A Systematic Review

**DOI:** 10.1155/2015/282405

**Published:** 2015-10-19

**Authors:** A. C. Martins, A. M. Rosa, E. Costa, C. Tavares, M. J. Quadrado, J. N. Murta

**Affiliations:** ^1^Centro Hospitalar e Universitário de Coimbra, Avenida Bissaya Barreto-Praceta Prof. Mota Pinto, 3000-075 Coimbra, Portugal; ^2^Faculty of Medicine, University of Coimbra, Rua Larga, 3004-504 Coimbra, Portugal

## Abstract

*Purpose*. This paper aims to review the morphological and functional characteristics of patients affected by familial amyloid polyneuropathy (FAP), with greater focus on type I and its progression after liver transplantation. We also analyse therapeutic options for the ophthalmic manifestations. *Methods*. The literature from 2002 through 2015 was reviewed, with a total of 45 articles studied, using the key terms related to amyloidosis and its therapeutic approaches. Information was collated, evaluated, critically assessed, and then summarised in its present form. *Pathophysiology and Treatment*. FAP results from mutation of the transthyretin gene, with Val30Met being the most frequent substitution. The symptoms are those typical of a sensorimotor autonomic neuropathy and can be halted with liver transplantation. Nowadays there are new medical therapies that delay the progression of the systemic neuropathy. However, there are still no options to avoid ocular disease. *Conclusion*. The main ocular manifestations in patients with FAP type I are amyloid deposition in the vitreous, dry eye, and secondary glaucoma. Despite liver transplantation, eye synthesis of amyloid persists and is associated with progressive ocular manifestations, which require continued ophthalmologic follow-up. New therapeutic strategies are therefore needed, particularly to target the ocular synthesis of the abnormal protein.

## 1. Introduction

Amyloidosis is a group of diseases characterised by deposition of amyloid, consisting of clumps of insoluble proteins at the level of the peripheral or central nervous system [1]. Its phenotype varies depending on the affected organs [2].

This condition may be primary in origin, very often hereditary and leading to familial amyloid polyneuropathy (FAP), or secondary to chronic inflammatory diseases and causing a sporadic form of the disease, senile systemic amyloidosis (SSA) [3, 4]. The latter is age related and has a milder clinical picture, mainly affecting the heart [5, 6].

FAP is a progressive autosomal dominant neurodegenerative disease, characterised by the accumulation of amyloid in the peripheral nerves and other organs, including the eye. It shows high phenotypic and genotypic heterogeneity, with incomplete penetrance and variable age of onset [7, 8].

It can be classified into three main types, according to the amyloid-forming precursor protein. The most common is transthyretin (TTR), and the others are apolipoprotein A-1 (ApoA1 ratio) and gelsolin (AGel) [9].

FAP originates from mutations of the TTR gene [10]. Over 100 different mutations have been described [1, 9]. The substitution of valine for methionine at position 30 of the TTR gene (Val30Met) is the commonest and the most studied mutation worldwide [11]. This mutation is responsible for the high prevalence of the disease in endemic areas, particularly the north of Portugal, Sweden, and Japan [6].

FAP can thus be classified according to its clinical characteristics and geographical origin [4]:

- Type I/Portuguese Type/ATTR: it is the most common type and primarily affects the lower limbs, with severe autonomic dysfunction. Portugal, Sweden, and Japan are endemic areas.
- Type II/ATTR: polyneuropathy starts in the upper limbs, with mild autonomic dysfunction; it is common in families in Switzerland and Germany.
- Type III/ApoA1: polyneuropathy, renal failure, and cranial neuropathy are also characteristic.
- Type IV/Type AGel: lattice corneal dystrophy type II is characteristic; it is more common in Finnish, Irish, American, and Japanese families; apart from polyneuropathy it is characterised by cutaneous hyperextensibility.

FAP has also been divided into three stages according to the progression of neuropathy [2]:

- Stage I: there is impairment of the lower limbs, without difficulty in walking.
- Stage II (after 5-6 years): there is impairment of the upper limbs and there is need for aid in walking.
- Stage III (after 10 years): there is total dependence.

## 2. Methods

We performed a systematic review of English- and Portuguese-language articles, restricted to studies published from 2002 through 2015, with a total of 45 articles related to ocular manifestations in FAP and the therapeutic options. We used terms such as amyloidosis, transthyretin, familial amyloid polyneuropathy, clinical trial, ocular manifestations, diflunisal, tafamidis, liver transplantation, small interfering RNA, antisense oligonucleotides, pharmaceutical company names, and other related terms, alone and in various combinations, as keywords. Incidence rates and availability of therapeutic approaches and their relative risks were assessed.

The materials were collated, evaluated, critically assessed, and then summarised in their present form.

## 3. Structure and Function of TTR

TTR is a protein circulating in the blood at a concentration of 20–40 mg/dL, forming a stable tetramer. It carries retinol and, in a lesser amount, thyroxine (T4) [1]. If not attached to TTR, retinol is filtered by the kidneys and excreted in urine [12]. The TTR gene is located on chromosome 18 (18q12.1) [6, 13]. When mutated, its tetramer conformation turns into monomers that aggregate and form amyloid deposits [1, 2]. TTR is also present in the cerebrospinal fluid and aqueous humour [5]. About 90% [11, 14] is synthesised and secreted by the liver and a small portion (<2%) may be synthesised by the brain's choroid plexus, the retinal pigment epithelium (RPE) [6, 11], and the small intestine [15].

## 4. Pathogenesis

The Val30Met substitution is the most frequent, and it is the only variant found in Portugal, Brazil, and Sweden [2]. So far, over 120 mutations in the TTR gene have been described [5]. Several in vitro studies have demonstrated that the TTR tetramer dissociation and consequent formation of amyloid fibrils are necessary processes to cause amyloidosis [6].

However, other mutations have been described, associated with milder phenotypes, such as Arg104His/Val30Met and Thr119Met/Val30Met, as well as mutations associated with more aggressive phenotypes, such as Leu55Pro [2]. TTR Val122Ile mutation is the most common pathogenic variant in the United States [9].

It was found that, in patients with Val30Met mutation, the circulating monomers are mostly wild-type; that is, they do not result from the mutation and are identical to those produced in senile systemic amyloidosis. This suggests that the mutant molecules are more unstable than the wild-type ones and therefore more prone to aggregation. However, the fact that circulating monomers are mostly wild-type could also simply result from the faster elimination of the mutant molecules [6].

Some authors suggest that the loss of peripheral nerve fibres that occurs in FAP, resulting in local ischaemia, is caused by endoneurial amyloid deposits [15]. Another possible mechanism to explain the toxicity of amyloid deposits is direct toxicity to cells [2].

It has been suggested that plasma TTR does not cross the blood-retinal barrier [14], despite evidence of progression of ocular manifestations, after liver transplantation. This reaffirms the belief that intraocular amyloid fibrils are not synthesised by the liver but locally at the RPE [11]. It is surprising, however, that the retinal deposits are mainly located in the inner layers rather than the layers closer to the RPE [11].

Other studies have suggested that amyloid deposits in the anterior chamber of the eye are produced by the pigmented ciliary epithelium, while vitreous deposition originates from the RPE [16].

## 5. History

FAP associated with TTR (TTR-FAP) was described in Portugal in 1939 and published in 1952 by Corinho Andrade, a Portuguese neurologist who studied a population of 74 patients from Póvoa de Varzim, a city in the north of Portugal [17, 18].

New cases were identified in Japan in 1968 and Sweden in 1976, and the disease can now be found worldwide [2].

The first TTR mutation causing FAP was described in 1985 on chromosome 18q11.2-q [15].

Liver transplantation is currently the only therapy that can halt the natural course of the disease, although there are other medical alternatives that may delay its progression. The first liver transplant for TTR-FAP was performed in 1990 on a male Swedish patient with type I FAP [19].

In 1995, in Coimbra, Portugal, Linhares Furtado performed the first liver transplant from a donor with FAP to a patient with metastatic liver disease. This type of transplantation has been known since then as sequential or “domino” transplantation [20]. Nowadays, livers from patients with FAP are even transplanted to patients with cirrhosis or carcinoma [14].

## 6. Epidemiology

TTR-FAP is considered a rare disease, given its low incidence of <1/200; the prevalence of the genetic mutation is 1 in 1 million [21], with an incomplete penetrance [15].

Despite that fact that it occurs throughout Portugal, Póvoa de Varzim and Vila do Conde are the cities which have the largest clusters of people with TTR-FAP V30M [16], with a prevalence of 1/538 [6, 22] to 1/1000 people and more than 500 families diagnosed [2].

About half of all cases of TTR-FAP do not have a family history and are designated as sporadic cases [2].

Families originating from endemic areas of Portugal and Japan usually have earlier onset of the disease with higher penetrance [5].

Women have a later onset of the disease than men (33.7 + 5.8 versus 29 + 6.4) [23].

In the US Caucasian population, the prevalence of Val30Met mutation in FAP patients is about 1 in 100,000. In Sweden, the frequency of heterozygosity is about 1.5%, with very low penetrance.

In the Afro-American, West African, and Hispanic populations, it was observed that the most frequent mutation is Val122I1e, with a prevalence of 3.0–3.9%, 5.0%, and 0.44%, respectively. This mutation's largest clinical expression is hypertrophic restrictive cardiomyopathy [2, 6].

## 7. Diagnosis

The lag time between the onset of symptoms and FAP diagnosis is usually 2 to 6 years [24]. FAP requires a biopsy and pathological exam to demonstrate amyloid deposits. Nerves, heart, kidney, colorectal mucosa (sensitivity 70–80%), abdominal fat aspirate, or salivary glands can be biopsied [21]. Tissues are stained with hematoxylin and eosin to reveal homogeneous eosinophil extracellular areas [2]. Because of its beta-pleated configuration, the amyloid substance stains with Congo red and has green birefringence, when viewed under polarised light [6]. Amyloid deposits are also stained by thioflavin s. Under specular microscopy it is possible to see the unbranched, thick parallel margins of the amyloid fibrils [2].

For a faster and more reliable diagnosis, genetic testing can be done to check for the TTR Val30Met mutation [21].

To rule out the presence of eye disease, the initial approach should include a full ophthalmological examination, including measurement of visual acuity, biomicroscopy with pupil and anterior chamber examinations, fundoscopic exam, and analysis of visual fields [2].

## 8. Clinical Manifestations

The clinical manifestations of FAP are highly variable. The homozygous phenotype resembles the heterozygote one in Val30Met mutation patients and is also indistinguishable from amyloidosis acquired by deposition of immunoglobulin light chains [25]. The average age for the onset of symptoms is around 33 but can vary from age 17 to 78. Approximately 80% of cases occur before age 40 and 85% of patients have a positive family history [22].

Initial symptoms are typically autonomic sensorimotor neuropathy, including paraesthesia, decreased thermal and pain sensitivity in the extremities and in the cornea [8], afferent ataxia, and autonomic dysfunction [1]. Neuropathy is usually symmetrical with focal distribution and centripetal progression [2, 16].

Extra central nervous system manifestations are the result of amyloid deposits in organs such as the eyes, heart, kidneys, and gastrointestinal system [6]. About ten years after FAP diagnosis and without treatment, the patient is at the final stage of the disease, suffering flaccid paralysis of the limbs, multiorgan dysfunction, and autonomic dysregulation [1].

Ocular manifestations are present in 10% of patients with TTR-FAP [9] and usually occur later during the course of the disease [22]. These symptoms correlate with neither the systemic symptoms nor the duration of the disease [11].

Kawaji et al. [26] described the case of a patient with ATTR Val30Met FAP without systemic manifestations and whose first and only manifestation was the amyloid deposit in the vitreous [26].

The main ocular manifestations present in FAP patients include [4, 7, 14]

- vitreous opacities;
- chronic open-angle glaucoma (COAG);
- abnormal conjunctival vessels (ACVs);
- keratoconjunctivitis sicca (KCS);
- loss of corneal sensitivity and neurotrophic corneal ulcers;
- anterior capsule opacity of the lens;
- retinal vascular changes;
- pupillary light-near dissociation;
- irregular pupil;
- optic neuropathy.

The amyloid deposition in the vitreous, with subsequent gradual decrease in visual acuity [2, 27], is almost pathognomonic of hereditary amyloidosis by mutation of the TTR gene, occurring either during the natural course of the disease or after hepatic transplantation [25]. This situation is more prevalent and occurs earlier in patients with Tir114Cis (100%) and Lys 54 mutations than in patients with variant Val30Met (24%) [28].

**Figure 1:**
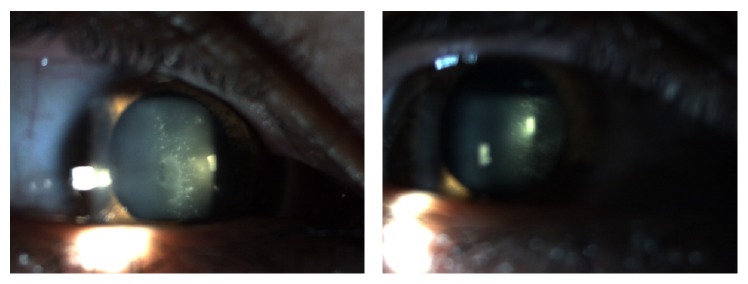
Man aged 42 underwent liver transplantation at age 27. Visual acuity decreased 3 years after pars plana posterior vitrectomy in 2005. There are amyloid deposits in the anterior vitreous, adhering to the lens posterior capsule [4].

Figure 1 shows the eye of a patient with FAP I 15 years after liver transplantation. We can see the vitreous deposits adhering to the posterior lens capsule, which is referred to as* pseudopodia lentis* [4].

In these patients, COAG is the leading cause of irreversible blindness [29].

The pathophysiological mechanisms responsible for the elevation of intraocular pressure (IOP) include perivascular amyloid deposition in conjunctival and episcleral tissues, intratrabecular deposition, and deposition of amyloid on the pupillary edge, which may precede glaucoma by months or years [4].

In patients with glaucoma, erythropoietin (EPO) is increased in the aqueous humor, exerting a protective effect on the photoreceptors, RPE, and ganglion cells. But the same is not seen in patients with glaucoma and FAP [29]. Therefore, in patients with FAP, substances with neuroprotective effect are scarce, which leads to the need for more aggressive treatments to preserve vision.

ACVs, described as red dots and segmental and fusiform dilatation of conjunctival vessels, afflict almost all patients during the disease. These changes result from liver synthesis of TTR, not from intraocular production, and consequently there is no progression after liver transplant, as expected [4].

Dry eye in FAP may be due to either autonomic neuropathy or amyloid deposition in the lacrimal gland [16], contributing to neurotrophic keratopathy and cornea perforation, which has been described in some cases [8]. Amyloid deposition in the cornea progressively lowers its sensitivity and damages the epithelium and stroma. Both situations contribute to the pathophysiology of dry eye, corneal epithelial injury, and parakeratosis [8].

Low or absent corneal sensitivity, spontaneous epithelial breakdown, and impairment of corneal healing characterise neurotrophic keratopathy (NK), a degenerative corneal disease that can threaten sight. Familial corneal hypoesthesia manifests itself by decreased corneal sensation, reflex tearing, blinking, and foreign body sensation [30].

Dosso and Rungger-Brändle [8] reported the case of a patient with FAP with bilateral corneal perforation who underwent bilateral penetrating keratoplasty (PK). Amyloid deposition in the cornea has a direct toxic effect by changing its sensory innervation and damaging the epithelium and stroma. Corneal amyloid deposition was also found after PK.

Intraocular production of mutated TTR leads to amyloid deposition in the anterior lens capsule that is often asymmetrical between the two eyes. This condition may impair spatial contrast sensitivity at all frequencies [18] and lead to early presbyopia in patients with FAP [16]. This is related on the one hand to a loss of lens elasticity and on the other to autonomic neuropathy, which affects the ciliary muscle accommodation [31].

Beirão et al. [31] found that 35 patients with FAP presented with presbyopia earlier than the normal population (32 versus 42 years) and required higher diopter addition. They also concluded that liver transplantation has no influence on the development of presbyopia.

Retinal changes occur in about 20% of FAP patients, normally as haemorrhages or cotton wool spots, and they are more prevalent in patients with Y114C mutation [32].

Kojima et al. [33] reported the case of a 59-year-old patient with FAP with choroidal vascular changes observed on indocyanine green angiography in the form of hyperfluorescent foci along the choroidal vessels.

**Figure 2:**
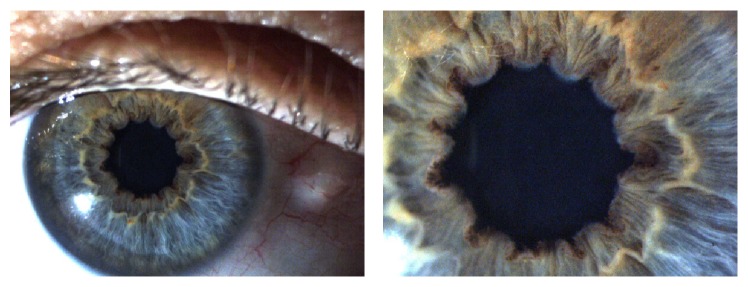
Multiple indentations of the pupillary edge and amyloid deposits in a 43-year-old patient with FAP 1, submitted to liver transplantation about 9 years ago [4].

Another ocular manifestation in patients with FAP is amyloid deposition at the pupillary edge, leading to peculiar indentations, as can be seen in Figure 2 [4].

There is also pupillary light-near dissociation, explained by the deposition of amyloid in the iris [11].

A rare cause of blindness in these patients is bilateral optic neuropathy. Hamann et al. [32] were pioneers in publishing a case of bilateral optic neuropathy after excluding other diagnostic hypotheses, such as vitreous opacity or glaucoma. It concerned a Portuguese male patient with FAP TTR Val30Met who presented with visual impairment. It was possibly caused by ischaemia secondary to amyloid deposition in small vessels, as well as impairment of autonomic self-regulation.

A study conducted in Japan [11] analysed 9 autopsied eyes and confirmed the presence of the aforementioned ocular manifestations. During the study, all patients showed ACV and pupil changes. Retinal changes were detected in 8 patients (21.6%), including haemorrhages (*n* = 4), cotton wool spots (*n* = 3), and peripheral neovascularisation (*n* = 1).

In 1997, Ando et al. [34] analysed 37 patients with FAP I in Japan for a period between 1 and 12 years. Among the most important ocular manifestations, ACVs had a prevalence of 75.5%, pupillary changes 43.2%, KCS 40.5%, and glaucoma and vitreous opacities 5.4%. Ocular manifestations appeared after liver transplantation, probably due to the intraocular production of mutant TTR [16].

## 9. Phenotypic Variants

Areas where FAP resulting from Val30Met mutation is endemic have a higher number of patients with a positive family history and earlier onset of the disease. However, no endemic cases have a late onset of the disease, over age 50, and there is a higher prevalence in men and milder symptoms [1].

Type I FAP can be divided into early onset FAP (before age 50) and late onset. A 2006 Portuguese study [7] compared the clinical differences between 86 patients with similar gender and geographic distribution: 43 patients with early disease and 43 with late disease. The early onset cases were more commonly associated with positive family history and with autonomic dysfunction. In the late onset cases, however, organ involvement and neuropathic pain were more frequent.

Studies on Portuguese parents and children [24] have found that the age of onset of the disease is higher in women, and the mother is responsible for the transmission of the disease in 60% of the cases.

## 10. Therapeutic Options

Therapeutic options in a patient with FAP depend on the stage of the disease and the patient's age. In patients with a positive family history, presymptomatic medical assistance is of utmost importance, since there are treatments available that halt the progression of the disease but do not reverse the already existing nerve damage [1].

Thus the therapeutic options available are

- orthotopic liver transplantation or
- pharmacologic treatment [35]:
  1. *tafamidis* and* diflunisal*: for stabilisation of the TTR tetrameric form;
  2. gene therapy with antisense oligonucleotides and RNA interference: to block TTR hepatic synthesis;
  3. doxycycline: to promote the clearance of amyloid fibrils.

Liver transplantation was initiated in 1990 and is currently the standard treatment for FAP and the only one able to change its natural history [36]. Liver transplantation removes the main source of mutated TTR, resulting in the fast decline of its concentration to levels around 1%. Thus it stops the progression of neurological symptoms [34] and promotes patient survival, as long as it is performed at an early stage [3, 6].

Before the era of liver transplantation, median survival of patients with type I FAP was about 10 years after disease onset. With liver transplantation it was possible to double this figure [34].

Liver transplantation replaces the mutant type with wild-type TTR. However, that does not apply to the cerebrospinal fluid or the eyes, which continue to produce the mutated form through the choroid plexus and the RPE, respectively [5]. Eye deposition of amyloid may occur from 4-5 years after the liver transplantation [14].

Rosa et al. [4] studied 20 eyes of 10 patients with Val30Met mutation and detected some major changes: dry eye (20%); vitreous opacities (20%), existing after transplantation and in some cases recurring after posterior vitrectomy; secondary glaucoma (20%); corneal nerve hyperplasia in 2 patients (20%); and pupillary abnormalities (10%).

Similar results were found in a clinical study [37] of disease progression in 22 patients, where the onset of glaucoma was reported in 3 (14%) patients, amyloid deposits on the pupil's edge were also found in 3 (14%) patients, and vitreous opacities were found in 1 (5%) patient.

In a Japanese observational study [34], 22 patients with TTR Val30Met mutation and 3 with Tir114Cis mutation (Tyr114Cys) were observed and it was found that 3 (12%) had glaucoma, 3 (12%) had amyloid deposits on the pupil edge, and 1 (4%) developed vitreous opacities.

Obayashi et al. [3] described the disease course in a 28-year-old patient, transplanted two years after the disease onset. The patient presented pupil amyloid deposition and vitreous opacities 10 years and 13 years, respectively, after the transplantation.

Beirão et al. [16] studied 2 groups, 32 transplanted patients and 32 nontransplanted ones. After 15 years, there was an increase of the ocular manifestations and an attenuation of the differences between the two groups. This study concluded that transplantation does not influence the deposition of amyloid in the iris, retinal amyloid angiopathy, or tear film instability. Schirmer's test, which evaluates aqueous tear film deficiency, was most commonly abnormal in the nontransplanted group (81% versus 56%). Greater prevalence of amyloid deposition in the lens, vitreous body, and glaucoma was observed in the transplanted patients. Chronologically, the first manifestation observed was dry eye, followed by deposition of amyloid in the iris and anterior capsule in the lens and VAC. The vitreous deposition and glaucoma appeared later. The last manifestation was retinal amyloid angiopathy.

Despite being a very useful therapeutic strategy, liver transplantation has all the limitations of an invasive procedure and requires immunosuppressive adjuvant therapy. It does not halt the progression of ocular, CNS, or cardiac clinical manifestations [6].

Not all patients are candidates for liver transplantation, and it is not recommended for patients over 65 years of age, those in an advanced stage of disease, or those with heart failure. Nor is it an option in patients with senile systemic amyloidosis due to the continuous deposition of wild-type TTR [6].

To date, over 1.500 liver transplants have been performed from living and dead donors in 19 countries [2, 6]. Portugal is the country with most transplanted FAP patients [16]. Each year, 120 liver transplants are performed in FAP patients worldwide [5].

### 10.1. Transplant in Sequence/in Domino

Sequence transplantation or “domino” is the use of a FAP donor liver for transplantation in patients with end stage liver disease to provide the receiver with a longer life free of symptoms and at the same time to reduce the shortage of organ donors [19, 38]. It assumes that FAP carrier liver is functionally and structurally normal despite the long-term production risk (8–10 years) of the mutated protein, which could theoretically lead to FAP in the recipient [17, 21].

The aim is to restore function in patients with hepatic failure, such as cirrhosis or liver cancer, and at the same time to solve the genetic defect in patients with FAP [15].

Although the development of amyloidosis and ocular manifestations has been described in the recipients of domino liver transplants, these manifestations appear at a late stage and do not constitute a contraindication for the procedure [20]. Tafamidis and diflunisal are recent pharmacological treatments [6] that stabilise the TTR tetramer and prevent its disassembly by protein denaturation, thus slowing the progression of the disease. They can change FAP's natural history, especially if used at an early stage [12].

Tafamidis meglumine is the first authorised and only available drug for TTR-FAP [2]. It binds selectively to TTR, stabilising its tetrameric structure and thereby reducing the amyloid monomers. Clinical trials tested a dose of 20 mg for 18 months [39] and have demonstrated its efficacy [12], with adverse effects similar to those in the placebo group. In 2011 tafamidis meglumine was approved by the European Medicines Agency for the treatment of FAP, being approved in Europe for the disease in its early stage [1, 6, 12] and in Japan at any stage [6].

Diflunisal is a nonsteroidal anti-inflammatory agent [12] that binds to TTR and prevents amyloid fibril formation. It acts on the wild variant of TTR [6]. According to clinical trials, the dose of 250 mg twice a day for 24 months stabilises FAP patients, reduces the neurological progression, and maintains the quality of life [39], without showing any adverse effects. This agent has however not been authorised yet.

New gene therapies revealed promising results in clinical trials [6]. Gene therapy has been developed to suppress the expression of variant TTR [6]. Its purpose is to silence the TTR gene with the use of antisense oligonucleotides and RNA interference. These components are in phase III.clinical trials [12, 24].

Various agents to increase the clearance of amyloid fibrils are currently under investigation. These studies are in the preclinical and clinical phases [36].

In mouse models, doxycycline has shown increased clearance of amyloid by disruption of the fibrils and by promoting its absorption. However, as yet there is no randomised trial to evaluate its role in TTR-FAP [35].

Thus, with the emergence of new treatment options, liver transplantation may be replaced by those less invasive strategies that have proven efficiency not only in FAP but also in senile systemic amyloidosis [6].

## 11. Treatment of Ocular Manifestations

### 11.1. Keratoconjunctivitis Sicca

For patients with severe dry eye disease, patients refractory to treatment with topical artificial tear, or patients with closure of the punctum [40], topical cyclosporine has been shown to be beneficial in patients with FAP after liver transplant, showing symptomatic improvement and improving the quality life. Cyclosporine reduces inflammation and improves the composition of the tear film through its inhibitory action on T lymphocytes and by increasing goblet cells in the conjunctival epithelium. Its topical use is not associated with adverse effects and it has a low systemic absorption [40].

Other therapeutic options for corneal epithelial injuries with vitamins, collagenase inhibitors, anti-inflammatory agents, prophylactic topical antibiotic, or bandage contact lenses are frequently inadequate or of transient efficacy. In severe cases, oral doxycycline, autologous serum, amniotic membrane transplantation, tarsorrhaphy, and a conjunctival flap are employed alone or in combination. However, successful modulation of the healing response is rarely accomplished [30].

Marta Guerra and João Quadrado [30] evaluated the response to treatment with* Cacicol*, a matrix regenerating agent (RGTA, polycarboxymethyl glucose sulphate), designed to mimic the heparan sulphates bound to corneal extracellular matrix proteins, protecting them from proteolysis and enabling growth factors and cytokines to act on the injured site.

They studied the response to treatment in 3 patients with FAP and refractory NK. After an average period of 33 days, there was full reepithelialisation, without recurrence of corneal ulcer in the follow-up of 2–7 months. The treatment regimen was 1-2 instillations a week after debridement of the edges of the ulcer. No systemic or local side effects were noticed and no pain or discomfort during drop instillation was reported.

### 11.2. Glaucoma

With liver transplantation, there was an increased survival of patients and consequently a higher prevalence of glaucoma and greater need for therapeutic intervention.

Continuous production of mutant TTR can explain the difficulty in reducing IOP.

In patients refractory to topical therapy, IOP is usually between 35.0 ± 9.0 mmHg, often with good control after surgery [41]. The most promising strategy seems to be trabeculectomy with mitomycin C, keeping IOP less than or equal to 20 mmHg [29].

A Portuguese study analyzed the progression of glaucoma in 44 patients with TTR-FAP, where 29.5% of the patients had IOP less than 20 mmHg with medical therapy and 69.2% required surgical treatment [42].

EPO, as mentioned earlier, was shown to have a protective effect on ganglion cells and has been proposed as a possible neuroprotective treatment strategy [29].

### 11.3. Vitreous Opacity

If there is severe loss of visual acuity or if the opacity does not allow a view of the fundus, vitrectomy should be performed. The 25-gauge vitrectomy has proved to be a safe therapeutic option [43], with improvement in visual acuity, and may be considered the treatment of choice in patients with glaucoma and who have already undergone trabeculectomy [44]. This induces less damage and inflammation due to the smaller conjunctival incision and is preferred in patients with KCS, since it does not affect mucin secretion [44]. Vitreous opacities may recur after vitrectomy as described by Rosa et al. [4] due to local production of amyloid fibrils. This intervention can induce open-angle glaucoma through several mechanisms. On the one hand, vitrectomy increases oxidative stress in the trabecular meshwork and, on the other hand, in the absence of vitreous, the amyloid aggregates reach the trabecular meshwork and Schlemm's channel more easily and are deposited in both structures.

Thus, studies suggest that the vitreous acts like a filter which retains the amyloid fibrils and prevents their progression to the trabecular meshwork [43]. Therefore, incomplete vitrectomy is a viable option, to delay the progression of glaucoma.

## 12. Discussion and Conclusion

TTR is primarily synthesised in the liver; however, it is doubtful whether liver transplantation is a factor in the resolution of eye disease given that intraocular amyloid formation is independent of liver synthesis, as shown by RPE autonomy in amyloid production. Furthermore, TTR is unable to cross the blood-retinal barrier [16, 34].

Still, liver transplantation is now the only therapeutic option able to alter the natural course of the disease, at least until gene therapies are approved [10, 37]. It dramatically increases FAP patients' survival and thereby increases the prevalence of ocular manifestations over time [26]. Genetic and environmental factors, as well as immunosuppression, may influence the presence of posttransplant clinical manifestations [5].

The main ocular manifestations reported in studies of patients with type I FAP are the deposition of amyloid in the vitreous, dry eye, and glaucoma. Although ocular manifestations rarely arise as a first symptom, they are often part of the clinical course and should lead to a suspected diagnosis in patients from endemic areas [26].

New therapeutic approaches plus a rigorous follow-up are now needed to ensure quality of life for these patients whose ocular manifestations limit their daily life [16, 34].

A new substance, RGTA, polycarboxymethyl glucose sulphate, was experimented and showed compelling effectiveness in corneal wound healing; it was well tolerated by all patients, showing that this approach might be an excellent solution to treat NK, even in this particular group of patients [30].

Presymptomatic genetic testing may be of value in increasing survival, since patients will be able to access treatment in useful time, before the disease progresses [45].

When the genetic diagnosis is done, it is advisable to carry out the first eye exam too. In asymptomatic patients, follow-up should be every two years and in symptomatic ones it should be annual [16]. When there are ocular manifestations, the frequency of consultation varies depending on disease stage: it should be annual for ACV, every six months for KCS, for indentations of the pupillary edge, and for anterior lens deposition, and every three months for glaucoma, vitreous deposition, and retinal angiopathy [16].

In conclusion, since the liver transplant does not treat the eye disease, new therapeutic strategies, possibly gene therapy, are required if we are to give these patients quality of life, so as to avoid invasive treatments and their adverse effects and also address the ocular symptoms.
